# Analyzing Declining Trends, Patient Demographics, and Complications in Total Elbow Arthroplasty: Nationwide Retrospective Data Analysis

**DOI:** 10.3390/jcm14051645

**Published:** 2025-02-28

**Authors:** Assil Mahamid, Fairoz Jayyusi, Lior Laver, Mohammad Haj Yahya, Gal Wolff, Ali Yassin, Eyal Behrbalk

**Affiliations:** 1 Department of Orthopedics, Hillel Yaffe Medical Center, Hadera 3820302, Israel; fairoz.jayyusi@gmail.com (F.J.); laver17@gmail.com (L.L.); mohammadqhy@gmail.com (M.H.Y.); galwolff6@gmail.com (G.W.); dr.yassin.a88@gmail.com (A.Y.); eyalb@hymc.gov.il (E.B.); 2 Rappaport Faculty of Medicine, Technion University Hospital (Israel Institute of Technology), Haifa 3200003, Israel

**Keywords:** inpatient outcomes, total elbow arthroplasty, National Inpatient Sample, healthcare utilization

## Abstract

**Background:** Total elbow arthroplasty (TEA) was initially introduced for end-stage rheumatoid arthritis but has since expanded to include osteoarthritis and complex distal humerus fractures, particularly in elderly patients. Over the past two decades, TEA utilization trends have fluctuated, with a recent decline attributed to advancements in disease-modifying antirheumatic drugs. Despite its benefits, TEA presents a high complication rate, necessitating further investigation into clinical outcomes, costs, and postoperative management. **Methods:** This retrospective cohort study analyzed TEA procedures from 2016 to 2019 using the National Inpatient Sample (NIS) database. Patients were identified via ICD-10 codes, with elective procedures included to ensure homogeneity. This study examined temporal trends, patient demographics, comorbidities, complication rates, length of stay (LOS), and hospitalization costs. Statistical analyses included chi-square tests, *t*-tests, and multivariate regression to assess associations between patient characteristics and outcomes. **Results:** A total of 4110 TEA procedures were analyzed, revealing a 16% decline in annual volume from 2016 to 2019 (*p* = 0.012). The cohort had a mean age of 65.99 years, with a predominance of female (75.3%) and White (72.6%) patients. The median LOS was two days, and median hospitalization costs were USD 78,473 (IQR: 56,935–115,671 USD). The most prevalent complications included mechanical loosening (12.5%), blood loss anemia (10.6%), cardiac complications (5.7%), and prosthetic-related pain (3.3%). Multivariate analysis identified hypertension, anemia, and respiratory disease as significant predictors of adverse outcomes. **Conclusions:** TEA utilization has declined, likely due to medical advancements in rheumatoid arthritis management. The procedure remains associated with substantial complication rates, particularly in trauma-related cases. Findings highlight the importance of patient optimization, surgical expertise, and postoperative monitoring to improve outcomes.

## 1. Introduction

Total elbow arthroplasty (TEA) was originally developed as a specialized orthopedic intervention for the management of end-stage rheumatoid arthritis (RA). Over time, its clinical indications have expanded to encompass additional severe elbow joint pathologies, including osteoarthritis and complex distal humerus fractures, particularly in low-demand patients, typically over 65 years of age. Moreover, TEA is occasionally employed for less prevalent conditions such as hemophilic arthropathy, juvenile idiopathic arthritis, and primary or metastatic neoplasms [1,2,3]. Nevertheless, contraindications such as neurological compromise affecting hand functionality, cognitive impairments (e.g., dementia), patient noncompliance, and open fractures must be carefully evaluated. Relative contraindications also exist for patients requiring weight-bearing functionality through the affected upper extremity [4]. Over the past two decades, the utilization of TEA has demonstrated variable trends. Earlier epidemiological studies reported an increasing incidence of the procedure, with an annual growth rate of approximately 6.4% [5]. However, more contemporary analyses have highlighted a significant decline, with TEA volumes decreasing by 33% between 2010 and 2018 [6]. This trend may be attributed to advances in disease-modifying antirheumatic drugs, which have markedly enhanced the medical management of RA [7]. Hospitalization metrics indicate that the median length of stay for TEA procedures is approximately two days [8], with substantial variability in associated costs. Reported estimates range from 16,300 USD ± 4000 USD to 51,970 USD, depending on variables such as geographical location, hospital system, type of charges reported, and insurance coverage. Additionally, the surgeon’s fees typically range between 1228 USD and 1842 USD [5,9]. Despite its therapeutic benefits, TEA is associated with a relatively high complication rate compared to other major joint arthroplasty procedures. Complications include implant loosening, infection, a major contributor to revision surgeries, and periprosthetic fractures, which account for approximately 12% of primary TEA failures. Less common complications include bushing wear, particularly with constrained designs, as well as ulnar nerve deficits and triceps dysfunction [10,11,12]. Postoperative care protocols remain heterogeneous, with significant variation in permitted ranges of motion, weight-bearing restrictions, and lifetime activity recommendations [13]. With the use of the National Inpatient Sample Database (NIS), the present study endeavored to further elucidate the clinical outcomes, complication rates, healthcare resource utilization, and postoperative management strategies associated with TEA, thereby contributing to a more comprehensive understanding of its evolving role in orthopedic practice.

## 2. Methods

### 2.1. Data Source

This retrospective cohort study utilized data from the Nationwide Inpatient Sample (NIS), a comprehensive administrative database that captures inpatient hospitalizations across the United States developed by The Healthcare Cost and Utilization Project (HCUP). Patients who underwent TEA were identified using specific ICD-10 procedure codes, as detailed in the Abbreviations. The study period extended from 1 January 2016 to 31 December 2019, representing the most recent data available within the NIS framework at the time of analysis.

### 2.2. Cohort Definition and Selection Criteria

Key outcomes analyzed in this study included temporal trends in TEA utilization, etiological distributions, patient demographic and clinical characteristics, the prevalence of comorbidities, rates and types of postoperative complications, duration of hospital stay, and total hospitalization costs.

### 2.3. Statistical Analysis

All statistical analyses were conducted using R version 4.4.1, incorporating survey weights, strata, and cluster variables from the NIS database via the survey package to ensure nationally representative estimates. Descriptive statistics were used to summarize patient and hospital characteristics, with continuous variables reported as means with standard deviations (SDs) or medians with interquartile ranges (IQRs) and categorical variables as counts and percentages. Group comparisons were performed using the chi-square test for categorical variables and the Wilcoxon rank-sum test for continuous variables. Multivariate survey-weighted logistic regression models were used to identify factors associated with postoperative complications, adjusting for patient demographics (age, sex, race/ethnicity), primary payer, clinical comorbidities, and hospital characteristics. Age was treated as a continuous variable, while other covariates were categorical, with reference categories set as White race, Medicare insurance, and small hospital bed size. Results were presented as adjusted odds ratios (ORs) with 95% confidence intervals (CIs), with statistical significance defined as *p* ≤ 0.05. Temporal trends in total elbow replacement procedures were analyzed using weighted linear regression, with the annual procedure volume as the dependent variable.

### 2.4. Ethical Consideration

This study was granted exempt status by the institutional review board, as the de-identified nature of the NIS dataset ensured compliance with ethical standards for human subject research. AI tools were utilized solely to improve the clarity, grammar, and style of the manuscript’s English language. They were not employed for data analysis, interpretation, or content generation.

## 3. Results

**Figure 1:**
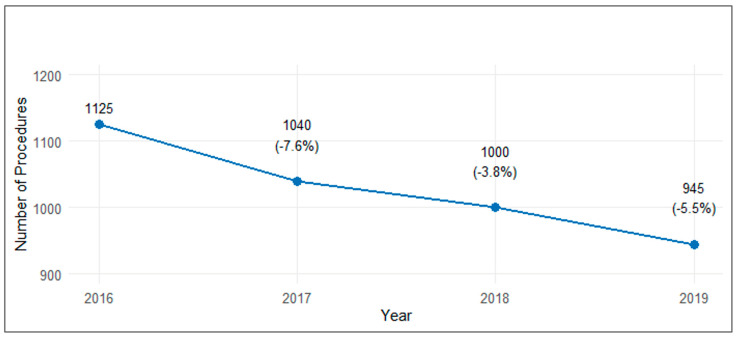
Trends in total elbow replacement procedures (2016–2019).

Between 2016 and 2019, there was a statistically significant downward trend in the number of TEA procedures (*p* = 0.012). Starting from 1125 procedures in 2016, the number decreased annually to 945 procedures in 2019, representing a total decline of 16% over the study period. The steepest year-over-year decline occurred in 2017 (−7.6%, −85 procedures), followed by more moderate decreases in 2019 (−5.5%, −55 procedures) and 2018 (−3.8%, −40 procedures). Linear regression analysis revealed a significant annual decrease of 58 procedures per year. Over the four-year period, the total number of procedures performed was 4110, with the annual proportion of procedures showing a gradual decrease from 27.4% in 2016 to 23.0% in 2019 (Figure 1).

Among 4110 patients who underwent TEA, the mean age was 65.99 ± 13.47 years, with a predominance of female patients (75.3%). The majority of patients were White (72.6%), followed by Hispanic (10.3%), Black (6.7%), and Asian (1.8%) patients. Hypertension was the most prevalent comorbidity (51.3%), followed by mental disorders (38.1%), dyslipidemia (37.2%), and type 2 diabetes (19.5%). Less common comorbidities included sleep apnea (12.9%), COPD (11.6%), chronic kidney disease (8.2%), and anemia (6.6%), while alcohol abuse (1%) and current smoking (0.5%) were rare. Most procedures were performed in large hospitals (57.2%) and urban teaching facilities (82.4%). The geographic distribution showed a higher concentration in the south (36.5%) and midwest (24.7%). Medicare was the primary payer for most cases (64.7%), followed by private insurance (23.2%) (Table 1).

The median total hospital charges for TEA were USD 78,473 (IQR: 56,935–115,671 USD). The mean length of stay (LOS) was 2 days, reflecting a relatively short postoperative hospitalization period (Table 2).

**Figure 2:**
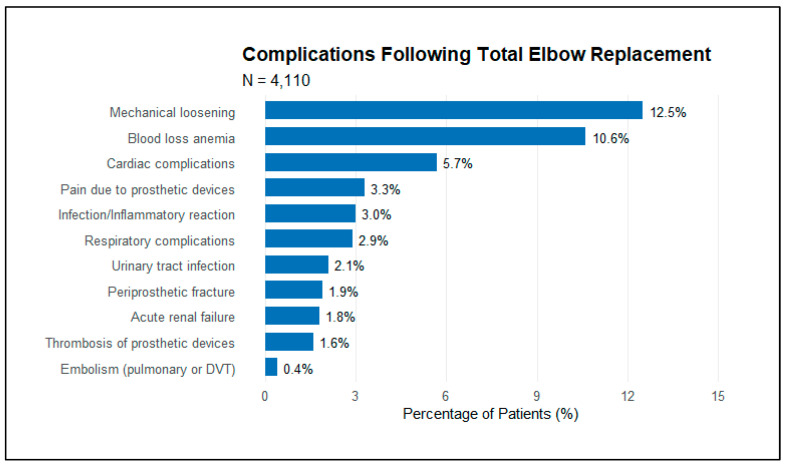
Postoperative complications of patients with total elbow replacement.

In the multivariate analysis of complications following TEA (Table 3), several significant associations were identified. Mechanical loosening was associated with younger age (OR, 0.98 per year; 95% CI, 0.97–1.00; *p* = 0.045), hypertension (OR, 2.49; 95% CI, 1.48–4.21; *p* < 0.001), and larger hospital size (OR, 1.62; 95% CI, 1.20–2.18; *p* = 0.001). Medical complications showed distinct risk patterns: both urinary complications and heart complications were associated with increasing age (OR, 1.07; *p* = 0.001 and OR, 1.08; *p* < 0.001, respectively). Respiratory complications demonstrated strong associations with multiple factors, including sleep apnea (OR, 7.44; 95% CI, 2.91–19.05; *p* < 0.001), chronic anemia (OR, 10.68; 95% CI, 3.24–35.17; *p* < 0.001), and female sex (OR, 5.85; 95% CI, 1.22–28.21; *p* = 0.027). Notably, chronic renal disease was a significant risk factor for both urinary tract infections (OR, 4.37; 95% CI, 1.22–15.70; *p* = 0.024) and inflammatory complications (OR, 4.93; 95% CI, 1.30–18.67; *p* = 0.019). Prosthetic pain showed a complex risk profile, with mental disorders (OR, 2.67; 95% CI, 1.19–6.01; *p* = 0.017) and COPD (OR, 2.93; 95% CI, 1.09–7.84; *p* = 0.032) increasing risk, while female sex was associated with lower risk (OR, 0.32; 95% CI, 0.14–0.77; *p* = 0.010) (Table 3 and Figure 2).

## 4. Discussion

This study provides a detailed analysis of TEA trends, demographics, and clinical outcomes using data from the Nationwide Inpatient Sample (2016–2019). It contextualizes TEA’s evolving role in orthopedic practice, with implications for patient management, surgical strategies, and healthcare resource allocation. 

Over the study period, a 16% reduction in annual TEA volume was observed, reflecting a significant shift in utilization patterns. This decline is largely attributed to the widespread use of disease-modifying antirheumatic drugs (DMARDs), which have mitigated joint destruction in rheumatoid arthritis. Historically, RA accounted for up to 80% of all TEA cases. However, global registry data indicate that this proportion has dropped to under 15% [1,14,15,16]. These findings affirm the success of biologics and combination DMARD therapies in reducing surgical interventions for inflammatory arthritis [7,17].

Simultaneously, trauma has become the leading indication for TEA, accounting for 40–50% of procedures globally [11,18,19]. This shift represents a reorientation of surgical priorities, with TEA now preferred over open reduction internal fixation (ORIF) for unreconstructedly fractures in elderly osteoporotic patients. Evidence suggests TEA provides superior outcomes compared to ORIF in these populations, cementing its role as the definitive treatment for complex fractures [3,20]. These trends demonstrate a broader redefinition of TEA’s utility within orthopedic surgery. Still, the most frequently affected osteoarthritis (OA) sites requiring arthroplasty are the coxo-femoral, knee, and shoulder joints, underscoring the widespread burden of degenerative joint disease in aging [21].

The demographic profile of TEA recipients reveals a predominance of older, White females, with a mean age of 65.99 years. This aligns with global trends, reflecting the higher prevalence of osteoporosis and fragility fractures among postmenopausal women [7,13]. Such demographics highlight the need for tailored surgical and perioperative strategies to address challenges specific to this population. Mechanical loosening, a complication observed in 12.5% of cases in this study, remains a significant concern, particularly in elderly patients with fragile bone quality.

The COVID-19 pandemic disrupted TEA utilization dramatically, with procedural volumes halving in regions such as England and Australia in 2020 [16,19]. Elective procedures were disproportionately affected, exacerbating delays in addressing both chronic arthropathies and acute fractures. These disruptions have highlighted existing disparities in healthcare access, particularly among socioeconomically disadvantaged populations. While procedural volumes are recovering, the long-term effects of these delays on patient outcomes require further investigation.

Institutional and surgical factors also significantly impact TEA outcomes. High-volume centers and experienced surgeons consistently report better results, underscoring the importance of procedural expertise and centralized care [7,15]. However, a substantial proportion of surgeons perform fewer than five TEAs annually, raising concerns about variability in outcomes. Centralization initiatives in the U.K. and Australia aim to address this issue by consolidating TEA procedures within high-volume centers, improving consistency and outcomes [16,19].

Comorbidities emerged as critical determinants of TEA outcomes in this study. Hypertension, anemia, and respiratory conditions were identified as significant contributors to perioperative risks and postoperative complications, including mechanical loosening and infections. Poorly controlled RA and osteoporosis further exacerbated these risks, emphasizing the importance of preoperative optimization. Metabolic conditions, such as diabetes, were associated with systemic complications, including pneumonia and urinary tract infections, while cardiovascular diseases heightened risks of perioperative complications [22,23,24]. These findings highlight the need for multidisciplinary care to optimize patient health prior to surgery.

Although our analysis included only ICD10 procedure codes of primary TEA, mechanical loosening emerged as the most frequently reported complication. However, this diagnosis cannot be accurately determined within the immediate postoperative period. This discrepancy may be attributed to miscoding during hospitalization, where the intended complication was likely prosthesis dislocation. Prosthesis dislocation is a recognized complication following TEA, though its reported incidence varies across studies. One study documented a 6% dislocation rate in their study of capitellocondylar elbow replacements. Similarly, another study reported a dislocation rate of approximately 2% in Souter-Strathclyde total elbow prostheses, while another study observed a 4.5% dislocation rate in long-term follow-up of TEA for distal humeral fractures. Although prosthesis dislocation is not the most prevalent complication, these findings underscore its clinical significance in the postoperative management of TEA [25,26,27].

And since we mentioned mechanical loosening, it is still considered a common cause for revision surgeries particularly in younger, more active patients and those treated in high-volume centers [17,19,28]. Although postoperative mechanical loosening is an infrequent occurrence, our multivariate logistic regression analysis identified hypertension as a significant risk factor for this complication. Consistent with the existing literature, hypertension has been linked to periprosthetic osteolysis and aseptic loosening of orthopedic implants by influencing bone remodeling and inflammatory pathways. The biological plausibility of this association is supported by evidence that chronic hypertension induces systemic inflammation and dysregulated bone metabolism, exacerbating the response to implant wear debris and ultimately leading to osteolysis and mechanical instability [29,30].

Blood loss anemia and cardiac complications also significantly impacted outcomes, underscoring the need for advanced perioperative management protocols. Trauma-related TEAs demonstrated higher complication rates than those performed for RA or osteoarthritis, reflecting the technical complexity and biological challenges of these cases [17,19]. Future studies should explore surgical approaches, fixation techniques, and postoperative protocols to improve outcomes for trauma-related TEAs. Infection remains a significant cause of morbidity and revisions, accounting for 15–35% of revisions globally [26,27]. Strategies such as antibiotic-laden cement and meticulous surgical techniques are critical to reducing infection rates. Nerve-related complications, though infrequent, are noteworthy, with the ulnar nerve particularly vulnerable during surgery [16,19].

Advances in prosthetic design have significantly influenced TEA outcomes. Linked prostheses, such as the Coonrad-Morrey and Nexel systems, remain the most widely used implants due to their superior stability and lower dislocation rates compared to unlinked designs. These systems achieve 10-year survivorship rates exceeding 80% [4,17,20]. Unlinked prostheses, while offering greater range of motion, are associated with higher instability and revision rates, limiting their widespread adoption [17,19]. Despite these advancements, persistent complications such as aseptic loosening and infection continue to challenge outcomes. Efforts to optimize implant design, cementation techniques, and surgical protocols are essential to mitigating these risks.

Our study has several notable strengths. First, it is the first analysis of TEA using the National Inpatient Sample (NIS) database, providing a nationally representative assessment of TEA utilization, complications, and outcomes. The inclusion of multiple years enhances the comprehensiveness of our dataset, ensuring a larger sample size than previous studies and improving the generalizability of findings. Second, we accounted for socioeconomic stratification and hospital characteristics, allowing for a more nuanced analysis of cost variations and healthcare resource utilization. By incorporating four years of data, we provide a broader overview of cost trends and length of stay, offering valuable insights into economic and institutional factors influencing TEA outcomes. Finally, this is the first study to analyze TEA outcomes using the ICD-10 coding system, ensuring relevance to contemporary clinical practice and healthcare reimbursement frameworks. This enhances the applicability of our findings and facilitates comparisons with future studies utilizing standardized coding methodologies.

This study has several limitations inherent to the use of the National Inpatient Sample (NIS) database in orthopedic surgery research. First, coding errors represent a significant challenge, as procedural and diagnostic codes rely on administrative data rather than direct clinical assessment. Misclassification, particularly between mechanical loosening and prosthesis dislocation, may lead to inaccuracies in complication rates. Second, the scope of hospitalization within NIS is limited to inpatient encounters, preventing the analysis of outpatient or perioperative factors that may influence surgical outcomes. This restriction may underrepresent complications that do not manifest during the initial hospital stay or those managed in ambulatory settings. Third, the lack of long-term follow-up limits the ability to assess critical postoperative outcomes such as implant longevity, delayed infections, or late-onset mechanical failures. Without longitudinal patient data, conclusions regarding long-term TEA performance and survivorship remain incomplete.

## 5. Conclusions

In conclusion, this comprehensive analysis underscores the need for continued innovation and refinement in TEA practices. Standardizing cost evaluations, centralizing care for complex procedures, and addressing systemic disparities are critical to ensuring the sustainable and effective utilization of TEA. Future research should focus on improving prosthetic design, optimizing perioperative care, and addressing demographic-specific needs to enhance outcomes and resource efficiency in this evolving field.

## Figures and Tables

**Table 1:** Patient demographics and characteristic of total elbow replacement patients.

Characteristic	N = 4110
**Age (years)** Mean (SD)	65.99 (13.47)
**Female**	619 (75.3%)
**Race**	
White	597 (72.6%)
Black	55 (6.7%)
Hispanic	85 (10.3%)
Asian	15 (1.8%)
Others	70 (8.5%)
**Type 2 Diabetes**	160 (19.5%)
**Hypertension**	422 (51.3%)
**Dyslipidemia**	306 (37.2%)
**Sleep Apnea**	106 (12.9%)
**Chronic Anemia**	54 (6.6%)
**Alcohol Abuse**	8 (1%)
**Mental Disorders**	313 (38.1%)
**Current Smoker**	4 (0.5%)
**Chronic kidney Disease**	67 (8.2%)
**COPD**	95 (11.6%)
**Hospital Size**	
Small	187 (22.7%)
Medium	165 (20.1%)
Large	470 (57.2%)
**Geographic Region**	
Northeast	134 (16.3%)
Midwest	203 (24.7%)
South	300 (36.5%)
West	185 (22.5%)
**Hospital Teaching Status**	
Rural	105 (2.6)
Urban nonteaching	620 (15.1)
Urban teaching	3385 (82.4)
**Primary Payer**	
Medicare	532 (64.7%)
Medicaid	46 (5.6%)
Private	191 (23.2%)
Other	53 (6.4%)

**Table 2:** Length of stay and total charges of total elbow replacement patients.

**Length of Stay (days) Median [IQR]**	2 [1–3]
**Total Hospitalization Charges (USD)** Median [IQR]	78,473 [56,935–115,671]

**Table 3:** Postoperative complications of patients with total elbow replacement.

Complication	%
Mechanical loosening	12.5
Blood loss anemia	10.6
Cardiac complications	5.7
Pain due to prosthetic devices	3.3
Infection/inflammatory reaction	3
Respiratory complications	2.9
Urinary tract infection	2.1
Periprosthetic fracture	1.9
Acute renal failure	1.8
Thrombosis of prosthetic devices	1.6
Embolism (pulmonary or DVT)	0.4

## Data Availability

Restrictions apply to the availability of these data. Data were obtained from HCUP and are available [https://hcup-us.ahrq.gov/ (accessed on 10 January 2025)] with the permission of HCUP.

## Abbreviations

HCUP	Healthcare Cost and Utilization Project
ICD-10	International Classification of Diseases, 10th Revision
TEA	Total Elbow Replacement
LOS	Length of Stay
RA	Rheumatoid Arthritis
NIS	Nationwide Inpatient Sample
SPSS	Statistical Package for the Social Sciences
